# Anaerobic fluorescent reporters for live imaging of *Pseudomonas aeruginosa*

**DOI:** 10.3389/fmicb.2023.1245755

**Published:** 2023-10-20

**Authors:** Caetanie F. Tchagang, Thien-Fah Mah, François-Xavier Campbell-Valois

**Affiliations:** ^1^Department of Biochemistry, Microbiology, and Immunology, Faculty of Medicine, University of Ottawa, Ottawa, ON, Canada; ^2^Centre for Infection, Immunity, and Inflammation, University of Ottawa, Ottawa, ON, Canada; ^3^Host-Microbe Interactions Laboratory, Center for Chemical and Synthetic Biology, Department of Chemistry and Biomolecular Sciences, University of Ottawa, Ottawa, ON, Canada

**Keywords:** *Pseudomonas aeruginosa*, anaerobic, biofilm, fluorescence microscopy, LOV, Fluorescence-Activating and Absorption-Shifting Tag (FAST), GFP, LucY

## Abstract

*Pseudomonas aeruginosa* thrives in the airways of individuals with cystic fibrosis, in part by forming robust biofilms that are resistant to immune clearance or antibiotic treatment. In the cystic fibrosis lung, the thickened mucus layers create an oxygen gradient, often culminating with the formation of anoxic pockets. In this environment, *P. aeruginosa* can use nitrate instead of oxygen to grow. Current fluorescent reporters for studying *P. aeruginosa* are limited to the GFP and related analogs. However, these reporters require oxygen for the maturation of their chromophore, making them unsuitable for the study of anaerobically grown *P. aeruginosa*. To overcome this limitation, we evaluated seven alternative fluorescent proteins, including iLOV, phiLOV2.1, evoglow-Bs2, LucY, UnaG, Fluorescence-Activating and Absorption-Shifting Tag (FAST), and iRFP670, which have been reported to emit light under oxygen-limiting conditions. We generated a series of plasmids encoding these proteins and validated their fluorescence using plate reader assays and confocal microscopy. Six of these proteins successfully labeled *P. aeruginosa* in anoxia. In particular, phiLOV2.1 and FAST provided superior fluorescence stability and enabled dual-color imaging of both planktonic and biofilm cultures. This study provides a set of fluorescent reporters for monitoring *P. aeruginosa* under low-oxygen conditions. These reporters will facilitate studies of *P. aeruginosa* in biofilms or other contexts relevant to its pathogenesis, such as those found in cystic fibrosis airways. Due to the broad host range of our expression vector, the phiLOV2.1 and FAST-based reporters may be applicable to the study of other Gram-negative bacteria that inhabit similar low-oxygen niches.

## 1. Introduction

*Pseudomonas aeruginosa* is a gram-negative opportunistic pathogen causing hospital-acquired diseases and infecting the lungs of cystic fibrosis patients. It is the most commonly isolated cystic fibrosis pathogen (Pressler et al., 2011; Winstanley et al., 2016) and is associated with worse clinical outcomes by accelerating the deterioration of pulmonary function (O’Brien and Fothergill, 2017). Therapeutic interventions against *P. aeruginosa* are complicated by its high level of resistance to multiple antibiotics and its ability to form biofilms (Wagner and Iglewski, 2008; Høiby et al., 2010).

Biofilms constitute a physical barrier to the host defense system (Magalhães et al., 2016). Moreover, *P. aeruginosa* cells within the biofilm are up to 1,000 times more resistant to antimicrobial compounds than their planktonic counterparts (Wagner and Iglewski, 2008; Hall and Mah, 2017). There is a steep O_2_ gradient within the cystic fibrosis airways caused by the accumulation of mucus that restrain O_2_ diffusion and the consumption of O_2_ by host cells and bacteria (Stutts et al., 1986; Worlitzsch et al., 1998, 2002). Thus, the oxygen concentration ranges from aerobic to anaerobic levels in cystic fibrosis lungs, and *P. aeruginosa* can thrive under all these conditions. In fact, although *P. aeruginosa* is an obligate aerobe, it can also grow anaerobically through denitrification using nitrate instead of O_2_ as an electron acceptor in the respiratory chain (Zumft, 1997; Shapleigh, 2013). Strikingly, the capacity of *P. aeruginosa* to form biofilms is superior in anoxia than in normoxia (Yoon et al., 2002), but tools to reliably study biofilms under low O_2_ are lacking.

Genetically encoded fluorescent reporters have revolutionized the imaging of dynamic processes in living cells, including in bacterial pathogens (Campbell-Valois et al., 2014; Bajunaid et al., 2020). The green fluorescent protein (GFP), with its high brightness and robustness to various environmental conditions, was instrumental in this endeavor. In addition, some variants, such as GFPmut2 (Iizuka et al., 2011), rapidly mature their chromophore, allowing for the monitoring of fast-paced cellular processes. However, GFP and related variants have drawbacks, notably their strict O_2_ requirement (Tsien, 1998). This property has limited their applications in living biofilms where O_2_ is low (Monmeyran et al., 2018).

By contrast, several alternative fluorescent proteins (FPs) are functional under oxygen-limiting conditions. However, to our knowledge, none of these FPs have been tested in *P. aeruginosa*. We thus selected seven FPs that spanned a wide range of excitation and emission spectra (Table 1). The first group evaluated was the light, oxygen or voltage proteins (LOV): iLOV (Chapman et al., 2008), phiLOV2.1 (Christie et al., 2012) and evoglow-Bs2, also known as EcFbFP (Drepper et al., 2007). These FPs are significantly smaller and mature faster than GFP, and they use flavin mononucleotide (FMN) as their chromophore (Chapman et al., 2008; Christie et al., 2012). One potential disadvantage of using these FPs is their low brightness (Kumagai et al., 2013). In addition, we selected LucY, which is brighter than many LOV and uses flavin adenine dinucleotide (FAD) as its chromophore (Auldridge et al., 2015). It is noteworthy that both FMN and FAD are abundant in the bacterial cytosol; thus, LOV and LucY spontaneously emit light in bacteria.

**Table 1:** Characteristics of the different fluorescent proteins used in this study.

FP acronym	Molecular weight (kDa)	Chromophore	Excitation (nm)	Emission (nm)	O_2_ dependence
GFPmut2	26.9	p-hydroxybenzylidene imidazole	480	511	Yes
iLOV	13.0	*FMN	450	495	No
phiLOV2.1	13.0		450	495	
evoglow-Bs2	15.8		448	496	
LucY	32.9	**FAD	276, 377, 460	530	No
FAST (green channel)	13.7	^TF^Amber	499	558	No
FAST (red channel)	13.7	^TF^Coral	516	600	No
UnaG	15.6	Bilirubin	498	527	No
iRFP670	34.5	Biliverdin	643	670	^ǂ^No

*FMN, Flavin Mono Nucleotide. **FAD, Flavin Adenine Dinucleotide. ^ǂ^As initially described in *Escherichia coli* (Rumyantsev et al., 2015). ^TF^Amber (HBR-3,5DM). ^TF^Coral (HBR-3,5DOM). GFPmut2, iLOV, phiLOV2.1, evoglow-Bs2 and LucY use an endogenous chromophore; FAST, UnaG and iRFP670 use an exogenous chromophore.

The Fluorescence-Activating and absorption-Shifting Tag (FAST) emits light when bound to a synthetic fluorogenic analog of 4-hydroxybenzylidene-rhodanine (HBR) (Plamont et al., 2016). The application of FAST in *P. aeruginosa* is promising because it was shown to outcompete the GFP in *Escherichia coli* biofilms (Monmeyran et al., 2018). UnaG and iRFP670 fluoresce when they form a complex with bilirubin (Kumagai et al., 2013) and biliverdin (Shcherbakova and Verkhusha, 2013), respectively. UnaG is characterized by its brightness and wavelengths similar to the GFP. Finally, the red-shifted emission of iRFP670 may address the auto-fluorescence of *P. aeruginosa* at shorter wavelengths, which is detrimental to the analyses of thick biofilms. It is important to note that bilirubin is not naturally present in *P. aeruginosa* and related bacteria, so it must be added to the growth medium. Similarly, free biliverdin is restricted in bacteria as its production is tightly dependent on iron availability. Therefore, it must be produced by an exogenous heme oxygenase that is co-introduced with iRFP670 into the bacterial host (Shcherbakova and Verkhusha, 2013).

Here, we report a panel of fluorescent proteins that successfully monitor *P. aeruginosa* in planktonic and biofilm cultures under anoxia. We demonstrate that phiLOV2.1 and FAST can be combined to track dual subpopulations of *P. aeruginosa* in microscopy experiments. This study provides tools that will be useful to researchers studying the behavior of *P. aeruginosa* when the concentration of oxygen is too low to use the GFP. The broad host range plasmid used to develop these reporters suggest that their use could be readily extended to other gram-negative bacteria.

## 2. Materials and methods

### 2.1. Construction of plasmids

pSMC21, a derivative of pSMC2 (Bloemberg et al., 1997), allowing the constitutive expression of the GFPmut2 under the *tacp* promoter, was used to derive several new plasmids. First, GFPmut2 was excised by PCR-mediated deletion mutagenesis with the High fidelity Phusion DNA polymerase (ThermoFisher) and recircularized with the T4 DNA ligase (New England Biolabs) to yield the resulting empty vector control pCFT1. Second, the coding sequences of seven alternative fluorescent proteins were amplified by PCR with the Phusion DNA polymerase and introduced independently into pCFT1 by combining PCR-mediated deletion mutagenesis and isothermal cloning with the NEB Gibson assembly master mix (NEB, #E2611), thus yielding pCFT2–8. The coding sequences of the fluorescent proteins iLOV, phiLOV2.1, evoglow-Bs2, LucY, FAST (Bio Basic Markham, ON) and UnaG (Integrated DNA Technologies, IA) were obtained by gene synthesis. iRFP670 and the heme oxygenase (HO) from *Bradyrhizobium* sp. ORS 278 were a kind gift from Vladislav Verkhusha (Shcherbakova and Verkhusha, 2013). The HO gene was cloned downstream of iRFP670 to generate a bicistronic gene. Both open reading frames were separated by two stop codons, a Shine-Dalgarno sequence AUCACCUCCUUA (Ma et al., 2002) and a 5-adenine spacer (Ringquist et al., 1992). The sequence of the cloned fragment in each resulting plasmid was confirmed by Sanger sequencing. The coding sequence of iLOV, phiLOV2.1, FAST and GFPmut2 were inserted by isothermal cloning into pUdO4a (Manigat et al., 2023), as described above, thus yielding pCFT3.2, pCFT4.2, pCFT7.2 and pCFT9.2 (Addgene plasmids #208806, #208807, #208808 and #208809 respectively). pUdO4a is a small plasmid (~2.8 kbp) that possesses strong transcription terminators L3S2P21, *thrLABC* and *arcA*, an ampicillin resistance gene and a BBR1 origin of replication, which can be maintained in *Pseudomonas* spp. (Antoine and Locht, 1992; Supplementary Figure S1 – plasmid maps). Table 2 and Supplementary Table S1 list the plasmids and primers used in this study.

**Table 2:** Plasmids used and generated in this study.

Plasmid name		Fluorescent protein	Antibiotic resistance	Reference
	pSMC21	GFPmut2	Ap, Cb, Km	Bloemberg et al. (1997)
pSMC21 derivatives	pCFT1	Empty vector (EV)	Ap, Cb, Km	This study
	pCFT2	evoglow-Bs2	Ap, Cb, Km	This study
	pCFT3	iLOV	Ap, Cb, Km	This study
	pCFT4	phiLOV2.1	Ap, Cb, Km	This study
	pCFT5	UnaG	Ap, Cb, Km	This study
	pCFT6	iRFP670	Ap, Cb, Km	This study
	pCFT7	FAST	Ap, Cb, Km	This study
	pCFT8	LucY	Ap, Cb, Km	This study
	pUdO4a	Empty vector (EV)	Ap, Cb	Manigat et al. (2023)
pUdO4a derivatives	pCFT9.2	GFPmut2	Ap, Cb	This study
	pCFT3.2	iLOV	Ap, Cb	This study
	pCFT4.2	phiLOV2.1	Ap, Cb	This study
	pCFT7.2	FAST	Ap, Cb	This study

Ap, Ampicillin; Cb, Carbenicillin; Km, Kanamycin.

### 2.2. Bacterial strains and growth conditions

The *Escherichia coli* strain DH10B was used for plasmid propagation. The wild-type *Pseudomonas aeruginosa* str. PA14 was used in the fluorescence experiments (Rahme et al., 1995). Unless otherwise stated, the culture medium was LB supplemented with ampicillin (100 μg/mL) or carbenicillin (100 or 200 μg/mL) for DH10B and PA14 transformants, respectively. Anaerobic culturing of PA14 was performed in LB supplemented with carbenicillin, and 100 mM KNO_3_ (LBN) to allow anaerobic respiration. Anaerobic planktonic cultures were incubated in anaerobic Hungate culture tubes with butyl rubber stoppers and screwcaps (Hungate, #CLS-4208). Biofilm anaerobic cultures were incubated in a BactronEZ chamber (Sheldon Manufacturing. Inc., Cornelius, OR) under an anaerobic atmosphere of 5% H_2_, 5% CO_2_, 90% N_2_ that contained fewer than 4.7 parts per million O_2_ per the manufacturer specifications. All incubations were performed at 37°C.

### 2.3. Growth curves under anaerobic conditions

PA14 strains harboring one of the pCFT plasmids were grown on LB agar carbenicillin plates. A single colony from each plate was used to inoculate 5 mL of LB carbenicillin. These cultures were aerobically grown for ~18 h with shaking (250 rpm), then subcultured for 5 h, and their optical density at 600 nm (OD 600) was adjusted to 1. The cultures were next transferred in the anaerobic chamber and diluted 1:100 in 5 mL LBN in anaerobic Hungate culture tubes. These sealed tubes were then incubated at 37°C for ~24 h with shaking (250 rpm) in an incubator located in a room with normal oxygen levels. Following this first incubation, the cultures were transferred back into the anaerobic chamber and diluted 1:10 in LBN. Finally, 100 μL of the resulting inoculates were dispensed in a flat black bottom 96-well plate (Greiner bio-one, #655096). The samples were overlaid with Johnson and Johnson ® oil, as described (Lam et al., 2018). Subsequently, the plate was sealed with an adhesive film (BioRad, #MSB1001), then wrapped with parafilm to prevent O_2_ exposure prior to exiting the anaerobic chamber. The plate was then incubated with medium shaking in a Hybrid Synergy H4 plate reader (BioTeK), where the absorbance at 600 nm was continuously tracked and recorded every hour for 24 h. The fluorescence signal of PA14 cells expressing the GFP (excitation: 480 ± 9.0 nm, emission: 511 ± 9.0 nm) was recorded at 0 and 24 h to ensure anoxia was maintained throughout the experiment. A sample of the 24 h culture was exposed to O_2_ for 2 h prior to the measurements to allow for the maturation of the GFP.

### 2.4. Fluorescence plate reader assay

PA14 cultures harboring pCFT plasmids were grown anaerobically in LBN at 37°C for 24 h in anaerobic Hungate culture tubes as described above. Depending on the fluorescent proteins assayed, the samples were treated as follows. First, PA14 expressing the flavin-binding fluorescent proteins iLOV, phiLOV2.1, evoglow-Bs2 and LucY were processed immediately. Second, PA14 carrying pCFT1 (empty vector) or pCFT5 (UnaG) were grown in LBN ± 10 μM bilirubin (Merck) and processed immediately. Third, after the anaerobic incubation in the regular anaerobic medium, 1.5 mL of PA14 cultures with pCFT1 (empty vector) or pCFT7 (FAST) were transferred to a microfuge tube with their cap tightly wrapped with parafilm inside the anaerobic chamber. These cultures were then centrifuged (2,400× *g*, 10 min) and resuspended in PBS ± 10 μM ^TF^Amber (HBR-3,5DM, The Twinkle Factory, Paris, France) (Li et al., 2017) as described (Streett et al., 2019). As described above, GFP-expressing cells were included in every assayed plate to confirm that anoxia was maintained during the experiment. The light emitted by the GFP-expressing cells exposed (or not) to O_2_ was measured along with the assayed FP at their optimal excitation and emission wavelengths (Table 1) using a Hybrid Synergy H4 Hybrid plate reader (BioTeK). The data were normalized according to the OD 600.

### 2.5. Live fluorescence imaging by microscopy

Experiments with PA14 planktonic cultures were carried out using the pSMC21 derivatives, whereas experiments with PA14 biofilm cultures were conducted with the pUdO4a derivatives. Aerobic PA14 cells carrying either phiLOV2.1, FAST or the empty vector were incubated in LB media supplemented with carbenicillin for 16 h to 18 h. These cultures were then diluted to an OD 600 of 1 and used as inocula for anaerobic cultures. A 1:1000 dilution of this inoculum was made for planktonic cultures that were subsequently grown anaerobically for 18 h in LBN. A 1:100 dilution of the inoculum was made for biofilm cultures grown anaerobically in a 35 mm glass-bottom dish (Cellvis, #D35-20-1.5H) underneath a 2% agarose pad in the anaerobic chamber. Biofilms were incubated in M63 minimal media supplemented with 1 mM MgSO_4_, 0.4% L-arginine, 100 mM KNO_3_ and carbenicillin (M63N) for 24 h. After the incubation, cells were prepared for imaging as follows. For planktonic cell imaging, the anaerobic cultures were centrifuged (2,400 × g, 10 min) and resuspended in M63N; for FAST imaging, this medium was supplemented with 5 μM ^TF^Coral (HBR-3,5DOM, The Twinkle Factory) (Li et al., 2017). For biofilm imaging, the culture medium was removed, and the attached cells were washed once in PBS before adding a 200 μL solution of M63N ± 10 μM ^TF^Coral. Both planktonic and biofilm cells were blanketed with a 2% agarose pad in a 35 mm glass-bottom dish (Cellvis, #D35-20-1.5H) for fluorescence imaging. The glass-bottom dish was then covered with a 35 mm glass-top cover (Cellvis, #D35-20-0-TOP) for differential interference contrast (DIC) imaging. To curtail O_2_ infiltration during imaging, the edges of the dishes were tightly wrapped in parafilm before exiting the anaerobic chamber for microscopy. It is noteworthy that although cells were incubated anaerobically and specific measures were taken to minimize O_2_ exposure during the imaging setup, the strict control of anoxia could not be warranted throughout the entire imaging process. Micrographs were acquired on a Zeiss LSM 880 AxioObserverZ1 confocal microscope equipped with an EC Plan-Neofluar 100×/1.3 oil M27 objective. Fluorescence was captured using the argon laser at 458 nm for phiLOV2.1 [excitation: 458 nm; emission: 466–544 nm (monocultures) and 466–522 nm (dual cultures)] and at 514 nm for FAST:^TF^Coral [excitation: 514 nm; emission: 555–722 nm (monocultures) and 586–722 (dual cultures)]. The photostability of iLOV and phiLOV2.1 in planktonic PA14 was evaluated by performing time-lapse acquisition every 30 s for 3 min under the conditions described above. All micrographs were collected with the ZEN software (Zeiss) and prepared with the ImageJ software (Schneider et al., 2012). The single cell quantitation of the fluorescence intensity from the planktonic micrographs was performed with ImageJ as previously described (Nigro et al., 2019). The total number of bacteria measured in these analyses was: 126 (empty vector, EV), 262 (LucY), 111 (UnaG), 218 (phiLOV2.1) and 168 (FAST:^TF^Coral), and were collected from four biological replicates.

### 2.6. Statistical analysis

For the plate assay experiments, three biological replicates are presented. The micrographs shown are representative of four biological replicates. The single-cell fluorescence intensity analyses were performed on micrographs of four biological replicates. Statistical analyses were computed with GraphPad Prism 8 (GraphPad Software, La Jolla, CA). Plate reader assay data are presented as mean ± standard deviation. The confidence level was set at 95% (*p* < 0.05).

## 3. Results

### 3.1. Construction of plasmid-borne fluorescent reporters

To study *P. aeruginosa* under anaerobic conditions, we picked the FPs iLOV, phiLOV2.1, evoglow-Bs2, LucY, FAST, UnaG and iRFP670 whose fluorescence is in principle independent of O_2_ (Table 1). The open reading frame of each of these proteins was cloned into the vector pCFT1 to generate pCFT2–8 (Supplementary Figure S1; Table 2). pCFT6 included a bicistronic operon of iRFP670 and HO to produce the biliverdin by heme degradation, a strategy that yielded functional iRFP670 in *E. coli* under anaerobic conditions (Shcherbakova and Verkhusha, 2013).

### 3.2. Anaerobic growth of PA is not impinged by the expression of FPs

**Figure 1:**
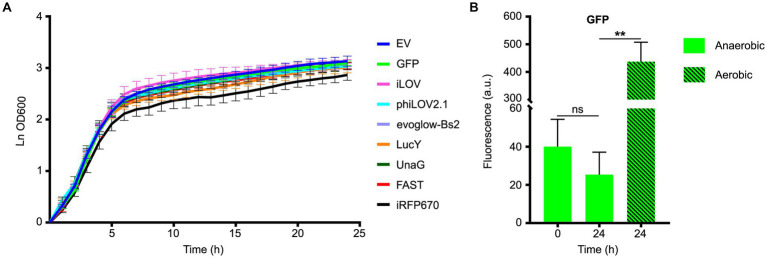
Fitness evaluation of *Pseudomonas aeruginosa* PA14 expressing different fluorescent proteins under anoxia. **(A)** Growth curves of PA14 carrying the plasmids pCFT1-8 and pSCM21 (Table 2), and incubated in LB supplemented with KNO_3_ and carbenicillin at 37°C under anaerobic conditions. The growth rate constant for each strain during the exponential phase was calculated and compared to the control empty vector (EV). The statistical significance of the change in exponential growth rate between the control EV and the strains expressing the fluorescent proteins was assessed by a two-tailed unpaired Student’s *t*-test (*p < 0.05*); none of the variations observed were significant. **(B)** The fluorescence of PA14 cells expressing GFP was measured at the beginning (0 h, anaerobic) and the end of the experiment (24 h, anaerobic), and after a subsequent exposure to oxygen (24 h, aerobic). Statistical analysis was performed using a paired Student’s t-test. Fluorescence was normalized with the OD 600, and the means with standard deviations as error bars are shown (ns = nonsignificant; *n* = 3).

To test the effects of the expression of the FPs on the growth of PA14, strains carrying the different plasmid were incubated in LB supplemented with carbenicillin and nitrate (LBN) under anaerobic conditions. Most strains carrying the plasmids containing the FPs grew similarly to the empty vector (Figure 1A). A notable exception was the strain carrying pCFT6, which contained iRFP670 and the HO, and appeared to grow at a slower rate. However, the statistical analyses of the growth rate during the exponential phase indicated this change was not significant (Supplementary Table S2). There was no increase in the fluorescence of the GFP between the beginning and the end of the measurements (Figure 1B), indicating that anoxia was maintained throughout the experiment. By contrast, exposure of the 24 h sample to O_2_ during 2 h increased fluorescence by approximately 20-fold, confirming that immature GFP had indeed accumulated during anaerobic growth (Figure 1B). Taken together, these results suggest that when cultured anaerobically, the viability and the growth of PA14 are unaffected by the expression of our collection of FPs, except perhaps for the iRFP670-HO bicistronic gene.

### 3.3. All FPs but iRFP670 are functional under anoxia

Since the fluorescent proteins had little to no impact on growth, we proceeded to compare their signal intensity. We assumed that their fluorescent signal would be representative of their overall behavior in PA14 and used it as a proxy that integrated their unique critical properties such as their expression level, maturation and brightness. This is a practical solution that circumvented the challenges arising from comparing a diverse set of proteins. Therefore, to evaluate the functionality of the FPs under anoxia, we first measured their fluorescence in PA14 using a plate reader assay.

**Figure 2:**
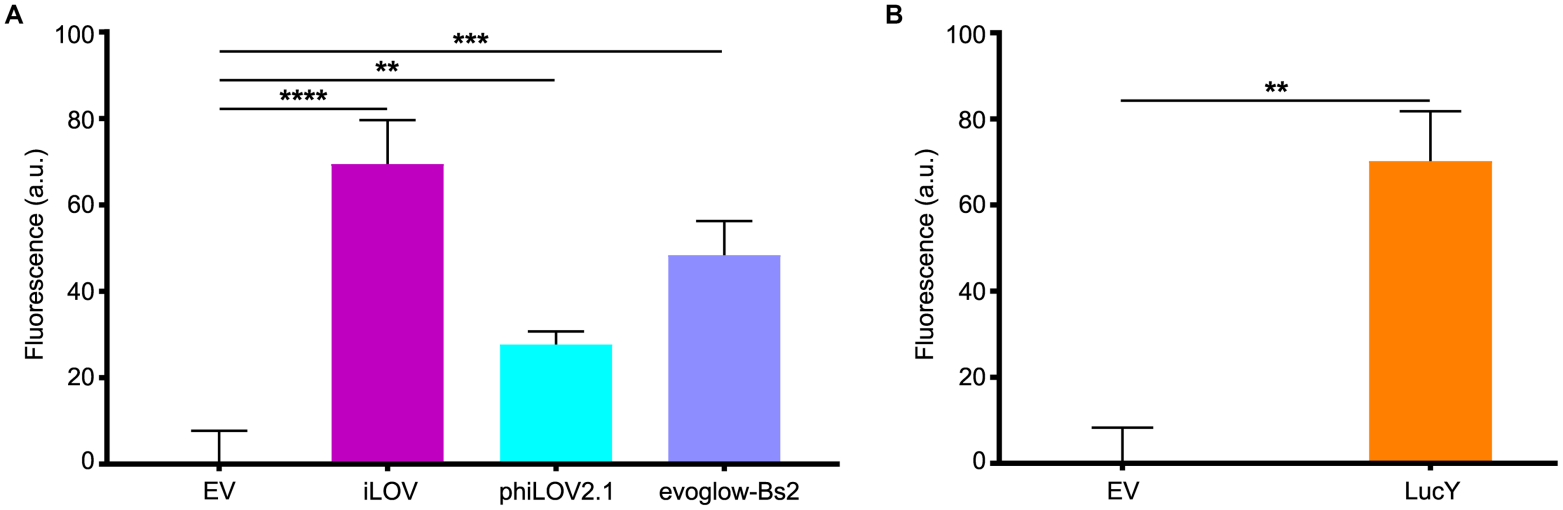
Fluorescence of PA14 expressing the flavin-based fluorescent proteins in anoxia. **(A)** Fluorescence of PA14 cells carrying plasmids allowing the expression of iLOV (pCFT3), phiLOV2.1 (pCFT4) and evoglow-Bs2 (pCFT2) compared with those carrying the empty vector (pCFT1). Fluorescence was recorded at excitation 450 nm and emission 495 nm. Statistical analysis was performed using one-way analysis of variance (ANOVA) followed by Bonferroni’s multiple comparisons test. **(B)** Fluorescence of PA14 cells carrying plasmids allowing the expression of LucY (pCFT8) expressed in PA14 cells compared with those carrying the empty vector (pCFT1). Fluorescence was recorded at excitation 460 nm and emission 530 nm. Statistical analysis was done using an unpaired Student’s *t*-test. Fluorescence was normalized with the OD 600, and the means with standard deviations as error bars are shown (**p < 0.01; ***p < 0.001; ****p < 0.001; *n* = 3).

**Figure 3:**
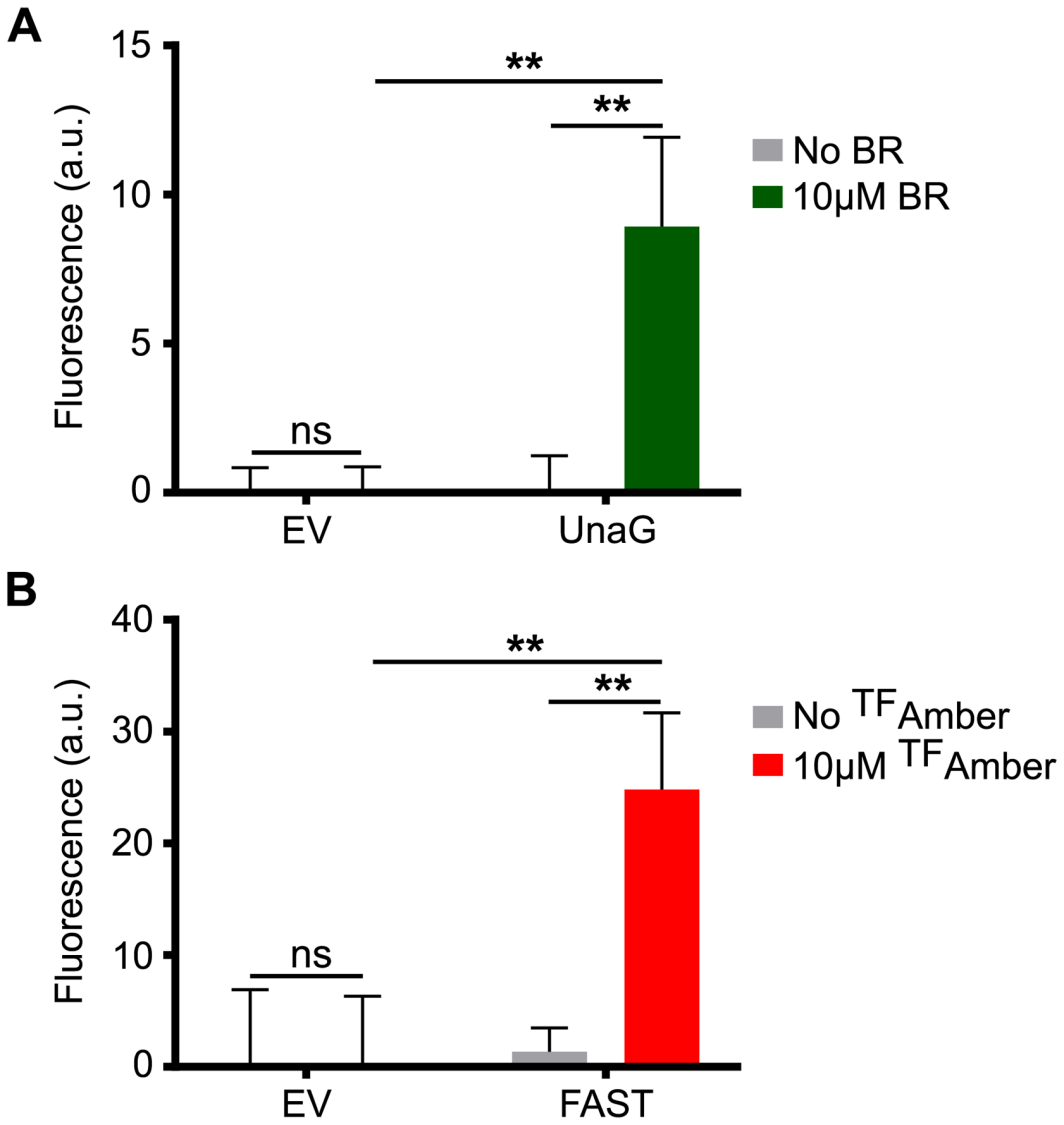
Fluorescence of PA14 expressing UnaG and FAST in anoxia. **(A)** Fluorescence of PA14 cells carrying plasmids allowing the expression of UnaG (pCFT5) compared with those carrying the empty vector (pCFT1) in absence and presence of 10 μM bilirubin (BR). Fluorescence was recorded at excitation 498 nm and emission 527 nm. **(B)** Fluorescence of PA14 cells carrying plasmids allowing the expression of FAST (pCFT7) compared with those carrying the empty vector (pCFT1) in the presence and the absence of 10 μM ^TF^Amber. Fluorescence was recorded at excitation 482 nm and emission 536 nm. In both panels, statistical analysis was executed using two-way analysis of variance (ANOVA) followed by Bonferroni’s multiple comparisons test. Fluorescence was normalized with the OD 600, and the means with standard deviations as error bars are shown (ns, nonsignificant; **p < 0.01; *n* = 3).

In this context, we grew planktonic PA14 cultures harboring the plasmids pCFT1–8 under anoxia and distributed them in 96-well plates under the anoxia-preserving conditions described above. PA14 expressing the LOV FPs showed a robust fluorescence signal compared to cells carrying the empty vector (Figure 2A), with iLOV showing the highest signal, followed by evoglow-Bs2 and phiLOV2.1. PA14 cells expressing LucY also displayed strong fluorescence relative to those carrying the empty vector (Figure 2B). Similarly, PA14 cells expressing UnaG (Figure 3A) and FAST (Figure 3B) exhibited a substantial fluorescence increase in the presence of 10 μM bilirubin and 10 μM ^TF^Amber, respectively. In contrast, the fluorescence of cells harboring the empty vector was negligible.

iRFP670 did not fluoresce when co-expressed with the HO in PA14 under anoxia (Supplementary Figure S2). In order to emit light, iRFP670 must form a complex with its chromophore biliverdin, which can be derived from heme in the presence of HO. Although this enzymatic reaction requires O_2_, previous data suggested that fluorescent protein related to iRFP670 can emit light in anaerobically grown *E. coli*, simultaneously expressing the HO (Rumyantsev et al., 2015). Our data indicated this is not possible in PA14. We reasoned that providing biliverdin, excess hemin or both in the growth medium might rescue the fluorescence. Unfortunately, these additives did not increase iRFP670 emission under anoxia. By contrast, iRFP670 fluoresced when co-expressed with the HO in aerobically incubated PA14, independent of exogenous additives (Supplementary Figure S2). Altogether, these data suggested that HO and O_2_ are required to yield enough biliverdin to produce functional iRFP670 in PA14. Hence, we did not investigate iRFP670 further. On the other hand, we pursued our investigation of the six FPs that emitted light under anoxia according to this plate reader assay.

### 3.4. Live fluorescence confocal microscopy imaging

As previously reported (Christie et al., 2012), we found phiLOV2.1 to be more photostable than iLOV (Supplementary Figure S3) and thus selected it for microscopic analyses of PA14. Next, we evaluated the other FPs to identify a suitable co-imaging partner for phiLOV2.1. Bacteria expressing LucY and UnaG were both visible in fluorescent microscopy (Supplementary Figure S4). The fluorescence of bacteria expressing LucY was bright and homogenous (Supplementary Figure S4A). By contrast, the fluorescence of bacteria expressing UnaG was heterogenous, with only a small subpopulation of strongly fluorescent cells (Supplementary Figure S4B). Subsequent single-cell analyses of the fluorescent intensity confirmed these observations (Supplementary Figure S4C). Thus, the use of UnaG in PA14 was complicated by the heterogeneity of its signal. Our attempts to resolve this issue failed and led us to discard UnaG.

**Figure 4:**
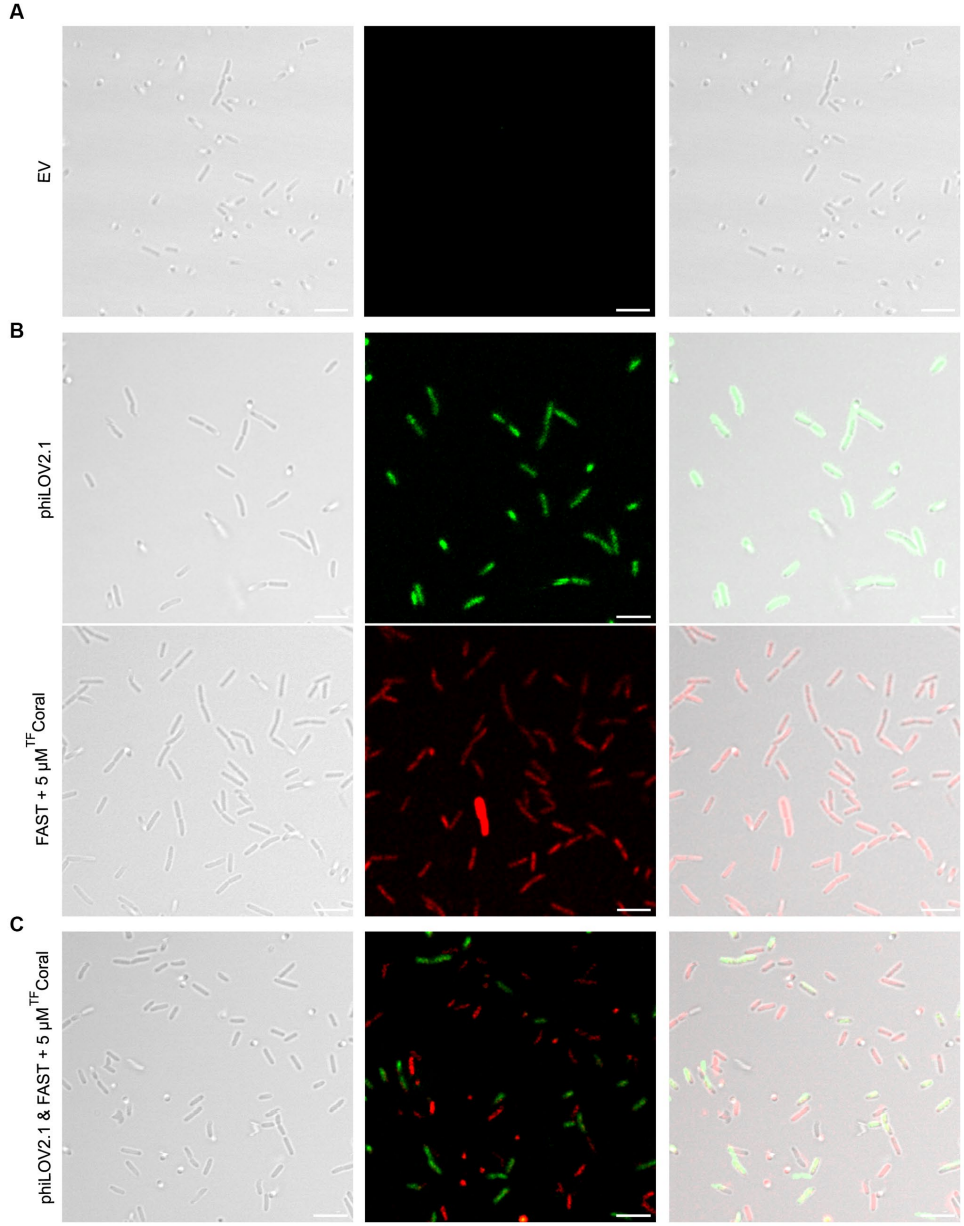
Fluorescence imaging of planktonic PA14 with phiLOV2.1 and FAST. **(A)** Micrograph of PA14 harboring the empty vector (pCFT1). **(B)** Micrograph of PA14 expressing phiLOV2.1 (pCFT4) and FAST:^TF^Coral (pCFT7) in the top and bottom panel, respectively. **(C)** Micrograph of a mix of PA14 cells expressing phiLOV2.1 or FAST:^TF^Coral (1:1 inoculation ratio). From left to right: differential interference contrast (DIC), single channel fluorescence and overlay (scale bar = 5 μm).

While the use of LucY seemed promising, it was not ideal for use in combination with phiLOV2.1 because their emission spectra overlap. The emission spectrum of FAST:^TF^Amber used in the plate reader assay also overlaps with phiLOV2.1. Hence, we turned our attention to FAST:^TF^Coral to cover the red region left unoccupied by the exclusion of iRFP670 as we reasoned it would combine well with phiLOV2.1. Indeed, planktonic PA14 cells carrying the empty vector were invisible (Figure 4A), whereas those expressing phiLOV2.1 or FAST:^TF^Coral were fluorescent (Figure 4B). The single-cell analyses of the fluorescent intensity showed that phiLOV2.1 yielded a homogenous and strong signal. By contrast, FAST:^TF^Coral resulted in a heterogenous and overall lower signal (Supplementary Figure S5). The heterogeneity of the signal observed with FAST:^TF^Coral is reminiscent of that of UnaG and may be attributable, at least in part, to their use of an exogenous chromophore. This hypothesis is supported by the homogenous signal of LucY and phiLOV2.1 who use an endogenous chromophore. Next, we tested the combined use of phiLOV2.1 and FAST:^TF^Coral to image dual cultures of *P. aeruginosa*. In brief, the strains used in monocultures were mixed and imaged. To avoid spectral bleed through, the span of wavelengths collected was narrowed for both fluorescent proteins. Once analyzed with these new acquisition parameters, the phiLOV2.1 and FAST:^TF^Coral subpopulations were distinguishable (Figure 4C). However, due to adjusting the acquisition parameters, the signal of both fluorescent proteins was dimmer than under the acquisition parameters used in the monocultures.

Preliminary microscopy observations indicated that light emitted by phiLOV2.1 and FAST:^TF^Coral decreased beyond detection limits in PA14 biofilms (data not shown). This decrease may be due to a reduction of the activity of the tac promoter or a reduction in the number of plasmid copies in biofilms. To address this pitfall, we subcloned the original tacp::phiLOV2.1 and tacp::FAST reporters into pUdO4a (Manigat et al., 2023). This synthetic plasmid harbors a broad host range BBR1 origin, which is functional in *Pseudomonas* spp. and differs from that used in pSMC21 (Supplementary Figure S1B; Antoine and Locht, 1992). In addition, the strong transcriptional terminators flanking the multiple cloning sites of pUdO4a may contribute to increasing gene expression (Manigat et al., 2023). Indeed, we found that the pUdO4a derivative pCFT4.2 expressing phiLOV2.1 produced higher fluorescence levels in planktonic PA14 than its pSMC21-derived counterpart pCFT4 (Supplementary Figure S5).

**Figure 5:**
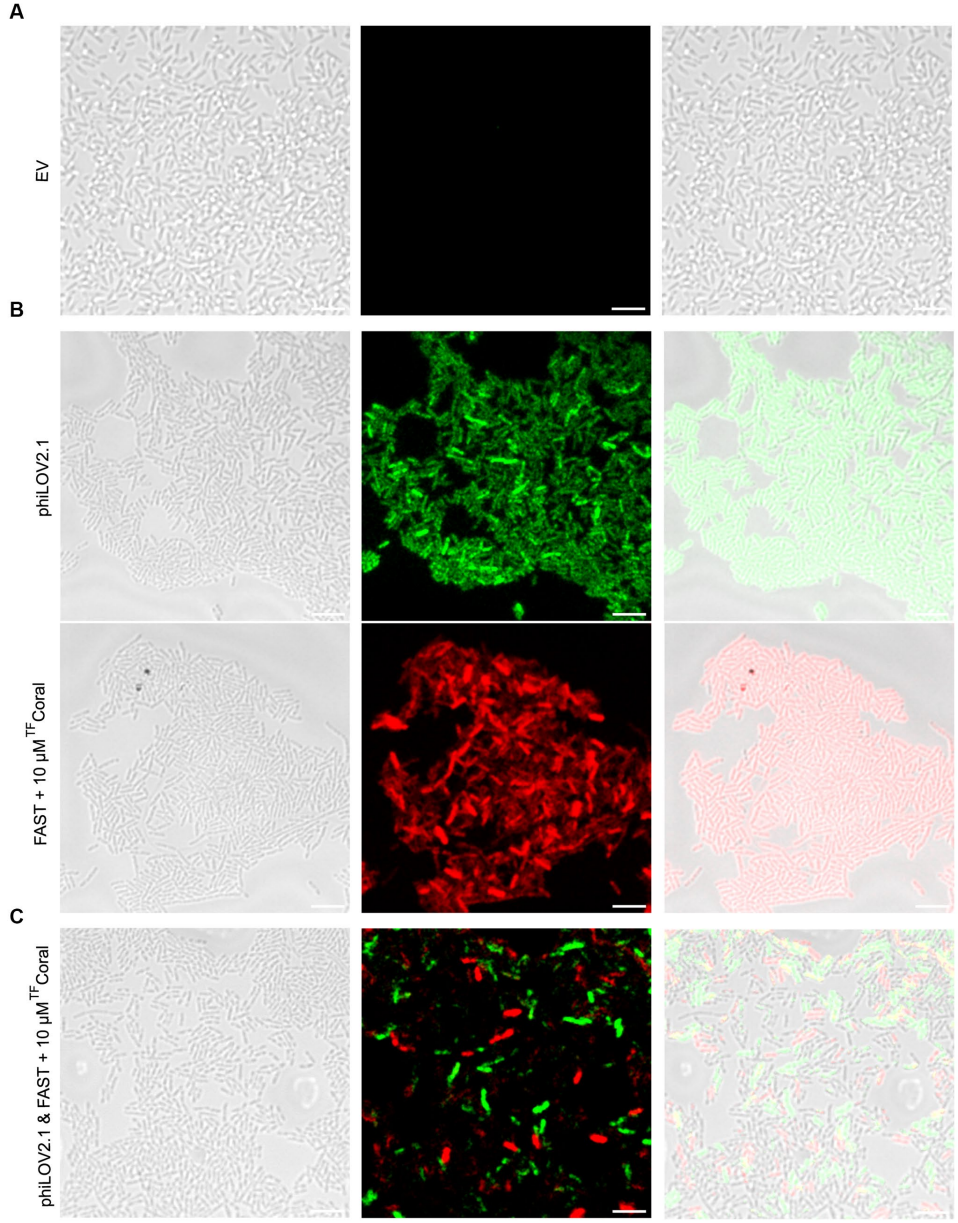
A new expression system allows fluorescence imaging of PA14 biofilms with phiLOV2.1 and FAST. **(A)** Micrograph of PA14 harboring the empty vector (pUdO4a). **(B)** Micrograph of PA14 expressing phiLOV2.1 (pCFT4.2) and PA14 expressing FAST:^TF^Coral (pCFT7.2) in the top and bottom panel, respectively. **(C)** Micrograph of co-cultured PA14 cells expressing phiLOV2.1 or FAST:^TF^Coral (1:1 mix ratio). From left to right: differential interference contrast (DIC), single channel fluorescence and overlay (scale bar = 5 μm).

Fortunately, the increased fluorescent signal of the FPs from the pUdO4a derivatives allowed the imaging of PA14 biofilms (Figure 5). Biofilm cultures carrying the empty vector were included as negative control (Figure 5A). In comparison with this control, biofilm monocultures of PA14 cells expressing phiLOV2.1 and FAST:^TF^Coral emitted light upon excitation at the expected wavelength (Figure 5B). Akin to the mixed planktonic cultures described above (Figure 4C), PA14 subpopulations expressing either of the two FPs were distinguishable in biofilm micrographs (Figure 5C). However, the signal distribution in the dual cultures appeared more scattered, with the appearance of a higher proportion of weakly non-fluorescent cells than in the monoculture. This could result from the adjustment of the acquisition parameters required for dual imaging, as described above. It is noteworthy that the optimal concentration of the ^TF^Coral chromophore was higher in biofilm than in planktonic cultures. This is likely due to the higher cell density in biofilms.

## 4. Discussion

In this study, we demonstrated that the fluorescent proteins iLOV, phiLOV2.1, evoglow-Bs2, LucY, FAST and UnaG allowed for the monitoring of live *P. aeruginosa* in plate reader assays under anaerobic conditions. In addition, we showed that phiLOV2.1, LucY and FAST:^TF^Coral are practical solutions for confocal microscopy. We characterized further the use of phiLOV2.1 and FAST:^TF^Coral and showed that they allowed for co-monitoring of dual subpopulations of *P. aeruginosa* by fluorescence microscopy. In summary, this study identified alternative fluorescent proteins for plate reader and microscopy assays when O_2_ levels are insufficient for the maturation of the GFP chromophore.

Our initial analyses included iRFP670. However, co-expressing this fluorescent protein and the heme oxygenase required to produce its biliverdin chromophore yielded no fluorescence under anaerobic growth. In mammalian tissue culture cells, biliverdin-binding fluorescent proteins such as iRFP670 could work under low oxygen conditions by scavenging biliverdin, which is ubiquitous in their cytosol (Ponka, 1999; Shcherbakova and Verkhusha, 2013). In several pathogenic bacteria, including *P. aeruginosa*, heme oxygenases are expressed under iron starvation conditions (Schmitt, 1997; Wilks and Schmitt, 1998; Zhu et al., 2000; Ratliff et al., 2001; Skaar et al., 2004; Wegele et al., 2004), limiting the natural production of biliverdin in the growth conditions used here. Therefore, a *Bradyrhizobium* heme oxygenase was introduced to increase the endogenous production of biliverdin (Shcherbakova and Verkhusha, 2013). However, in anaerobically grown *P. aeruginosa*, these heme oxygenases did not yield sufficient biliverdin to produce a detectable iRFP670 signal. This issue was not resolved by exogenous biliverdin, probably because of its lack of membrane permeability or accumulation in the cytosol. Conversely, the same strain grown in normoxia fluoresced. This suggests that cytosolic iRFP670 can function in *P. aeruginosa*, but the paucity of intracellular biliverdin precludes its use under anoxia.

UnaG has been used under aerobic and anaerobic conditions in *Bacteroides thetaiotaomicron, Bacteroides ovatus* and *E. coli* by adding bilirubin in the growth media (Chia et al., 2020, 2021). Here, we observed that *P. aeruginosa* expressing UnaG was also fluorescent when grown in the presence of bilirubin. Nevertheless, the signal was heterogeneous, with only a small fraction of the population emitting a high fluorescent signal. When averaged over the whole population in the plate reader assay, the signal of UnaG seemed inferior to the LOV proteins. Poor membrane permeability (Hancock and Woodruff, 1988) and efflux pumps, such as MexAB-OprM (Michea Hamzehpour et al., 1995; Plesiat et al., 1997), may contribute to limiting the amount of bilirubin available to UnaG in the cytosol of *P. aeruginosa*.

Regarding the LOV fluorescent proteins, our data were congruent with previous studies. The signal of iLOV was the strongest, whereas the signal of phiLOV2.1 was the weakest, and the signal of evoglow-Bs2 was intermediate (Buckley et al., 2015; Mordaka and Heap, 2018; Streett et al., 2021). However, the photostability of phiLOV2.1 (Christie et al., 2012) likely explains its superior performance in fluorescence microscopy of *P. aeruginosa*, which has also been observed in *Clostridium* species (Buckley et al., 2016). Although the fluorescence intensity of LucY was similar to iLOV as reported (Drepper et al., 2007; Auldridge et al., 2015), we did not investigate LucY in depth because phiLOV2.1 was a more practical option to use in combination with FAST. Indeed, the efficiency of FAST:HBR as fluorescent reporters has been previously described in several microorganisms, including *E. coli* (Monmeyran et al., 2018; Streett et al., 2019)*, Clostridium acetobutylicum* (Streett et al., 2019), *Listeria monocytogenes* (Peron-Cane Id et al., 2020) and in the archaea *Methanococcus maripaludis* (Hernandez and Costa, 2022). Likewise, we showed that FAST:HBR is suitable for monitoring *P. aeruginosa* in plate reader assays (^TF^Amber) and by confocal light microscopy (^TF^Coral). Taken together, these data suggest that phiLOV2.1 and FAST:HBR are currently the best candidates for imaging dual cultures of *P. aeruginosa* under anaerobic conditions.

The dual imaging of *P. aeruginosa* subpopulations with phiLOV2.1 and FAST:^TF^Coral was straightforward in planktonic cells. Upon switching to biofilms, we observed a substantial reduction in the fluorescence signal that was detrimental to imaging. We hypothesized that this phenomenon could stem from the downregulation of the promoter or a reduction of the number of copies of the plasmid when *P. aeruginosa* formed biofilms. This first generation of reporters was derived from pSMC21, a historical *P. aeruginosa* expression plasmid (Bloemberg et al., 1997; Cowan et al., 2000). It previously worked to monitor biofilm in aerobic conditions with the bright GFPmut2 (Davey et al., 2003). The low brightness of phiLOV2.1 and FAST:^TF^Coral revealed previously hidden suboptimal performance of pSMC21 as an expression vector. We, thus, switched these reporter genes to a novel plasmid called pUdO4a. This plasmid is maintained by a BBR1 origin, its sequence is fully known, much smaller than that of pSMC21, and its multiple cloning site is flanked by strong transcription terminators that may contribute to increasing the expression levels of a gene of interest. Indeed, the brightness of *P. aeruginosa* harboring the pUdO4a derivatives pCFT4.2 (phiLOV2.1) and pCFT7.2 (FAST:^TF^Coral) allowed the imaging of individual cells in the biofilms. Moreover, it also distinguished the two subpopulations in dual-culture biofilms. It is noteworthy that during co-imaging, the span of wavelengths collected was narrowed to avert signal overlap. This put some cells under the threshold of detection, thereby unveiling the limitation in the compatibility of phiLOV2.1 and FAST:^TF^Coral. Fluorescent proteins with brighter emission and more widely separated emission peaks would likely improve the imaging performance. Nevertheless, this study paves the way to monitoring dual *P. aeruginosa* populations in a variety of genetic background and environmental conditions. The broad host range of the BBR1 origin suggests that the second generation of pCFT reporters derived from pUdO4a could be seamlessly transferred to several other gram-negative bacteria.

This study identified six fluorescent proteins that are functional under anoxia in *P. aeruginosa*. However, their brightness is only a small fraction of that of the GFP under aerobic conditions. Therefore, discovering brighter and complementary O_2_-independent fluorescent proteins will drive future development by improving our capacity to monitor multiple *P. aeruginosa* subpopulations. For example, three brighter variants of phiLOV2.1 were recently described – phiLOV3, mini-GFP1 and mini-GFP2 (Babakhanova et al., 2022; Liang et al., 2022). Secondly, FAST derivatives such as FAST2 and tdFAST2 have improved brightness in their HBR-bound state (Tebo et al., 2018). Moreover, frFAST binds a different chromophore, which shifts the fluorescence emission palette toward the farred (Li et al., 2020). Alternative self-labeling proteins not tested here, such as SNAP_f_, CLIP_f_ and Halo-tag, yielded excellent anaerobic reporters (Los et al., 2008; Sun et al., 2011). Finally, pUdO4a and its derivatives described above provide an excellent platform to expand the fluorescent tools for studying microbes under oxygen-limited conditions in a variety of environmental and physiological settings.

## Data availability statement

The raw data supporting the conclusions of this article will be made available by the authors, without undue reservation.

## Author contributions

CFT contributed to the design of the study, performed the experiments, analyzed the data, and wrote the manuscript. FXCV and TFM obtained the funding, designed the study, and critically revised the manuscript. All authors approved the submitted version.
