# Supplementary material for: Anaerobic fluorescent reporters for live imaging of Pseudomonas aeruginosa
Source: Front Microbiol. 2023 Oct 20;14:1245755. doi: 10.3389/fmicb.2023.1245755 (PMC10623331; doi:10.3389/fmicb.2023.1245755)
Supplement: Supplementary file 1 [file Data_Sheet_1.pdf]

Supplementary Material

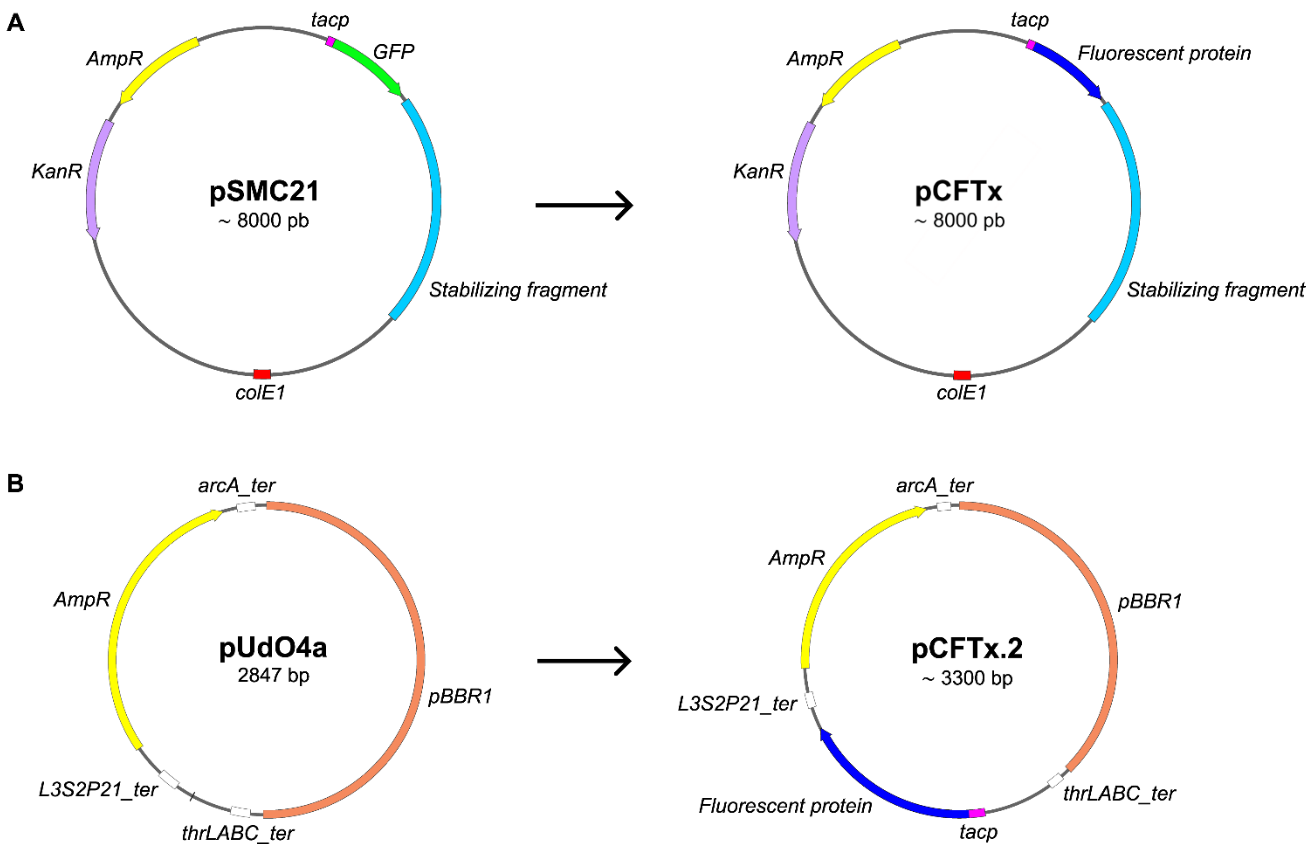

Supplementary Figure S1. Plasmid maps of pCFTx and pCFTx.2. **(A)** pCFTx derived from pSMC21, where the GFP has been removed or replaced by the fluorescent proteins listed in Table 1. **(B)** pCFTx.2 derived from pUdO4a where tacp::GFPmut2, tacp::iLOV, tacp::phiLOV2.1 and tacp::FAST were subcloned. The maps are not to scale and were prepared using SnapGene Viewer v.6.2.2.

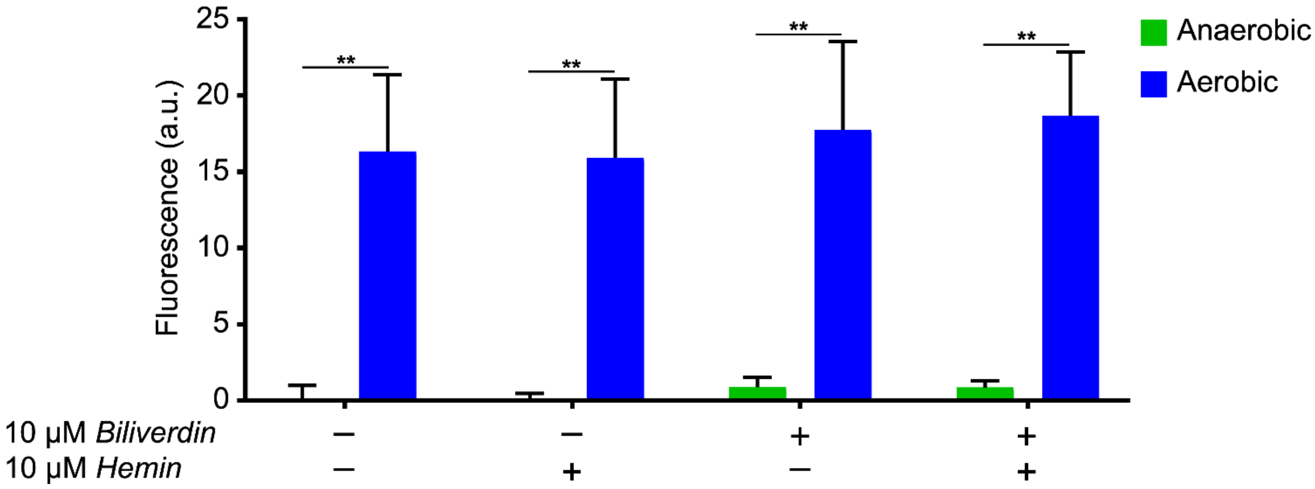

Supplementary Figure S2. Comparison of fluorescence level of *P. aeruginosa* PA14 expressing iRFP670 in normoxia versus anoxia. Fluorescence of PA14 cells expressing iRFP670 was recorded at 643 nm excitation and 670 nm emission wavelengths in the presence or absence of 10 µM biliverdin and 10 µM hemin. Fluorescence was normalized to OD600. Statistical analysis was computed using two-way analysis of variance (ANOVA) followed by Bonferroni's multiple comparisons test. The means with standard deviations as error bars are shown (**, $P < 0.01$; n = 3).

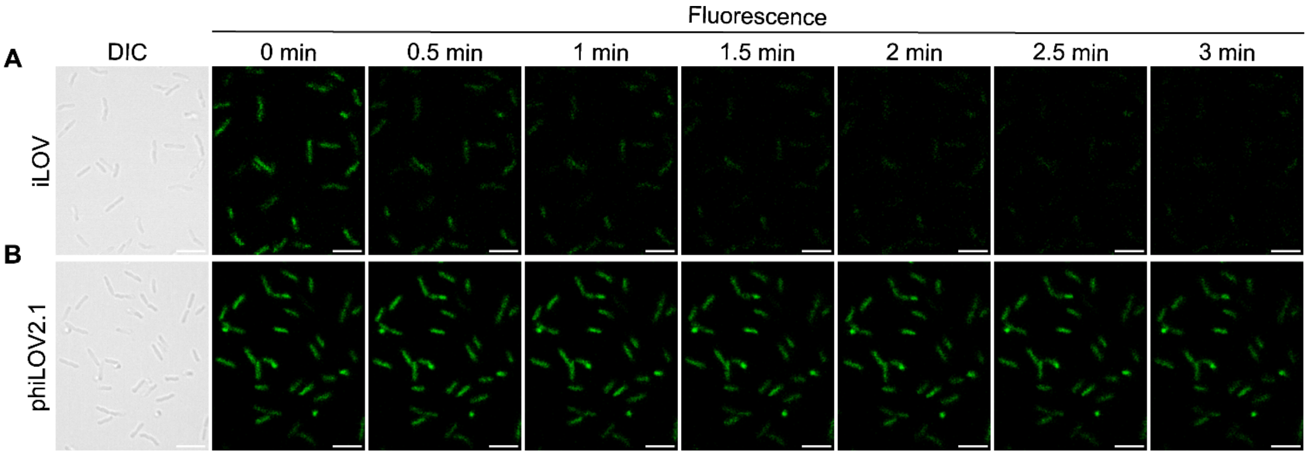

Supplementary Figure S3. Comparison of photostability between iLOV (pCFT3) and phiLOV2.1 (pCFT4) using time-lapse imaging of *P. aeruginosa* PA14 cells. Micrographs were acquired every 30 seconds for 3 minutes at excitation 458 nm and emission 505 nm. Far-left panels are differential interference contrast (DIC), and the others are single channel fluorescence (scale bar = 5µm).

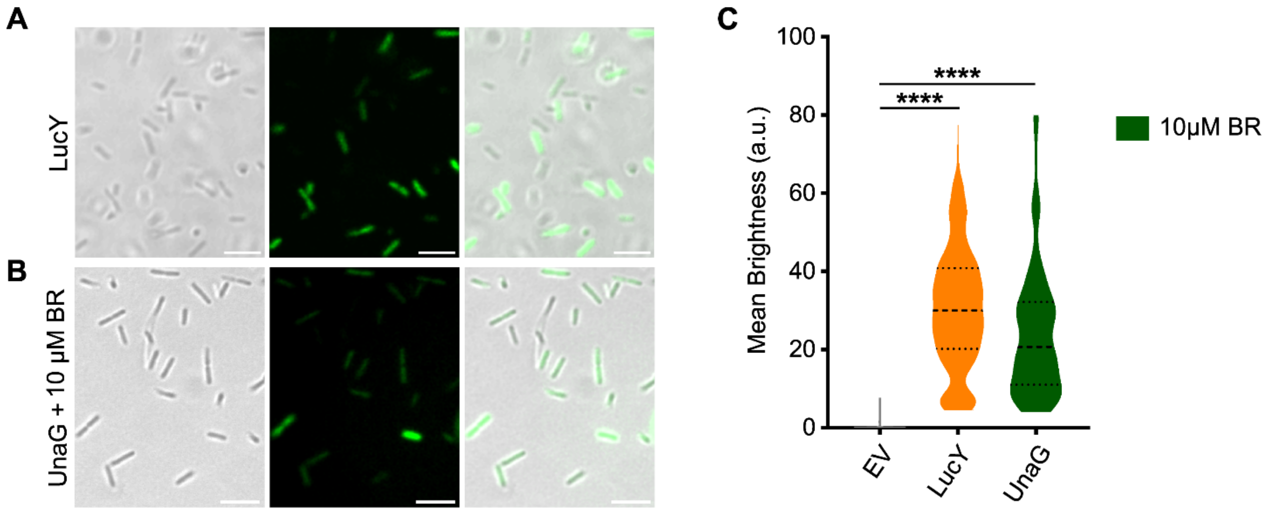

Supplementary Figure S4. Fluorescence of LucY and UnaG in anaerobic planktonic cultures of *P. aeruginosa* PA14 cells. **(A)** Micrograph of PA14 expressing LucY (pCFT8) recorded at excitation 488 nm and emission 558 nm. **(B)** Micrograph of PA14 expressing UnaG (pCFT5) in the presence of 10 µM bilirubin (BR) recorded at excitation 488 nm and emission 539 nm. From left to right: differential interference contrast (DIC), single channel fluorescence and overlay (scale bar = 5µm). **(C)** Single-cell fluorescence quantification of LucY and UnaG compared to EV, the empty vector (pCFT1) used as the control for background fluorescence. Mean brightness intensities were measured with ImageJ and statistical analysis was performed using an unpaired Student's t-test (****, $P < 0.0001$; n = 4).

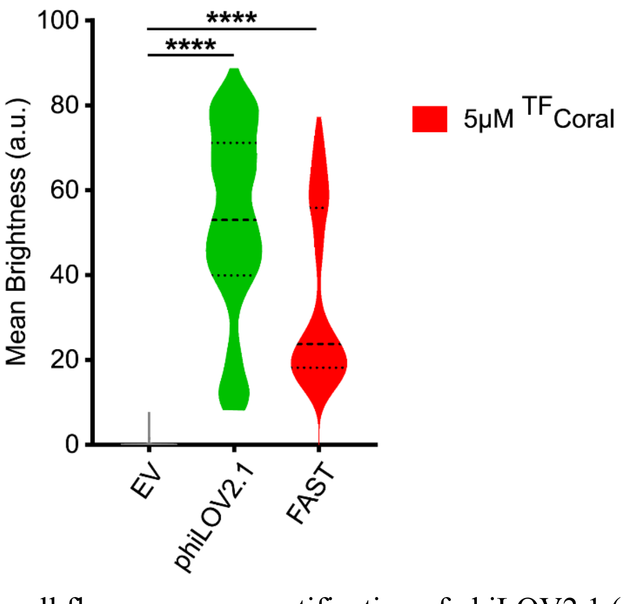

Supplementary Figure S5. Single-cell fluorescence quantification of phiLOV2.1 (pCFT4) and FAST (pCFT7) compared to EV, the empty vector (pCFT1) used as the control for background fluorescence. Mean brightness intensities were measured with ImageJ and statistical analysis was assessed using an unpaired Student's t-test (****, $P < 0.0001$; n = 4).

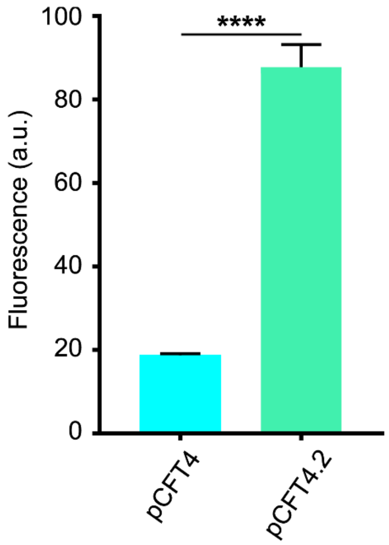

Supplementary Figure S6. PA14 carrying pCFT4.2 produced higher phiLOV2.1 fluorescence than pCFT4. Fluorescence of PA14 expressing phiLOV2.1 in two different plasmids background. Fluorescence was recorded at excitation 450 nm and emission 495 nm and normalized to OD600. Statistical analysis was performed using an unpaired Student's t-test. The means with standard deviations as error bars are shown (****, $P < 0.0001$; n = 3).

Table S1: List of primers used in this study

Long name	Primers sequences
pSMC21_rev_ctr	GTCTGGACATATGTATATCTCCTTCTTAAATCTAGAG
pSMC21_fwd_ctr	AGATATACATATGTCCAGACCTGCAGCAATG
pSMC21_rev	ATGTATATCTCCTTCTTAAATCTAGAG
evoglowBS2_fwd	TTTAAGAAGGAGATATACATATGAAAGCATCTTTCCAG
evoglowBS2_rev	ATTGCTGCAGGTCTGGACATTCAGTTACTCGAGCAGCTTTTC
pSMC21_fwd	CTGAATGTCCAGACCTGCAGCAATG
phiLOV2.1_fwd	TTTAAGAAGGAGATATACATATGAAAATCGAGAAATCGTTC
phiLOV2.1_rev	ATTGCTGCAGGTCTGGACATTCAGTTACACGTGATCAGACCC
iLOV_fwd	TTTAAGAAGGAGATATACATATGAAAATCGAGAAGAACTTC
iLOV_rev	ATTGCTGCAGGTCTGGACATTCAGTTACACATGATCCGAACC
UnaG_fwd	TTTAAGAAGGAGATATACATATGGTGGAAAAATTCGTCGGCAC
UnaG_rev	ATTGCTGCAGGTCTGGACATTCAGTTATTCGGTGGCGCGCCG
iRFP670_fwd	TTTAAGAAGGAGATATACATATGGCGCGTAAGGTCGATC
iRFP670_rev	TTTTATTCCTCCATTTCAGTTAGCGTTGGTGGTGGGC
heme_fwd_670	CCAACGCTAACTGAAATGGAGGAATAAAAAATGTTGGACCGAGTTGATTCCAA TCACGTC
heme_rev	ATTGCTGCAGGTCTGGACATTTAGTCAGCGGCCGAGCCCCGC
pSMC21_fwd_heme	CTAAATGTCCAGACCTGCAGCAATG
pUdO4a_noMCS_fwd	CGACACGGTCTCTGAGAG
pUdO4a_noMCS_rev	CGAAGACACTCGGCTCTTC
pUdO4a_FP_orf_revseq_fwd	CGAAGAGCCGAGTGTCTTCGCGGTAACGACGTCCAGAC
pUdO4a_FP_orf_revseq_rev	TTCTCTCAGAGACCGTGTCGCACTTAACATTATGCTGAGTGATATC

Table S2: Comparison of growth rates of *P. aeruginosa* PA14 expressing different fluorescent proteins in anoxia with EV (empty vector) as the control.

	EV	GFP	iLOV	phiLOV2.1	evoglow-Bs2	LucY	UnaG	FAST	iRFP 670
Slopes	0.43	0.43	0.45	0.42	0.44	0.4	0.42	0.42	0.38
P values	-	1.00	0.52	0.86	0.73	0.48	0.8	0.87	0.16
